# A Comparison of the Outcomes of Simultaneous Bilateral and Unilateral Mobile-Bearing Unicompartmental Knee Arthroplasties: An Analysis of Data from the National Joint Registry of England, Northern Ireland and Isle of Man

**DOI:** 10.2106/JBJS.OA.25.00292

**Published:** 2026-02-03

**Authors:** Hasan Raza Mohammad, Andrew Judge, David W. Murray

**Affiliations:** 1 Nuffield Department of Orthopaedics, Rheumatology and Musculoskeletal Sciences, University of Oxford, Nuffield Orthopaedic Centre, Oxford, United Kingdom; 2 Barts Bone & Joint Health, Blizard Institute, Barts and The London School of Medicine and Dentistry, Queen Mary University of London, London, United Kingdom; 3 Musculoskeletal Research Unit, Bristol Medical School, University of Bristol, Level 1 Learning and Research Building, Southmead Hospital, Bristol, United Kingdom

## Abstract

**Background:**

Unicompartmental knee arthroplasty (UKA) is an effective treatment for unicompartmental end-stage knee arthritis. Simultaneous bilateral UKAs for patients with bilateral knee arthritis can reduce costs, number of anesthetics, and overall rehabilitation time. It is unknown how the long-term outcomes of unilateral and simultaneous bilateral UKAs compare.

**Methods:**

In total, 1,939 unilateral and 1,939 simultaneous bilateral medial mobile-bearing UKAs (n = 3,878) from the National Joint Registry were propensity score matched. Kaplan-Meier and Cox regression were used to compare implant survival, revision indications, and mortality.

**Results:**

The 10-year implant survival in the simultaneous bilateral group was 92% (95% confidence interval [CI] 90-94) and in the unilateral group was 90% (95% CI 88-92). The simultaneous bilateral group had a lower revision risk (hazard ratio [HR] 0.73, p = 0.01). Revisions for pain were lower in the bilateral group (0.5% vs. 1.2%, p = 0.01). There were no differences in patient mortality. Subgroup analyses found similar trends in 10-year implant survival and revision risk with both cementless (simultaneous bilateral 98% CI 95-99; unilateral 95% CI 91-98; HR 0.66, p = 0.27) and cemented fixation (simultaneous bilateral 91% CI 89-93; unilateral 90% CI 88%-92%; HR 0.85, p = 0.28).

**Conclusions:**

Simultaneous bilateral UKAs had better 10-year implant survival and similar mortality to compared with single-unilateral UKAs. For patients with severe symptomatic bilateral unicompartmental knee osteoarthritis, simultaneous bilateral UKAs could be considered to be a safe and effective procedure, particularly as only one operation and postoperative recovery is required.

**Level of Evidence:**

Level IV. See Instructions for Authors for a complete description of levels of evidence.

## Introduction

Unicompartmental knee arthroplasty (UKA) is an effective treatment option for end-stage knee arthritis confined to one compartment. UKA has advantages over total knee arthroplasty (TKA) including a less invasive procedure, reduced mortality, and better postoperative function^1-3^.

One fifth of patients presenting for a knee replacement suffer from bilateral knee arthritis and are destined to undergo contralateral surgery within a few years of the primary operation^4-6^. Although there are several studies in the literature studying the outcomes of simultaneous bilateral TKAs, there is a paucity of evidence about simultaneous bilateral UKAs. This is important as TKA has higher risks of complications and mortality when simultaneous bilateral operations are performed^7,8^. UKA on the other hand may be better suited for simultaneous bilateral procedures given its minimally invasive approach allowing faster recovery and lower morbidity. Existing UKA studies only look at short-term outcomes and are limited by power.

An increasing number of surgeons are implanting simultaneous bilateral UKAs for patients with bilateral knee arthritis to lower costs, reduce anesthetics, address waiting lists, and prevent further functional decline^9^. The most commonly used UKA is the Oxford UKA (Zimmer Biomet, Swindon, UK). Many surgeons using the Oxford UKA have changed from using cemented to cementless fixation, as this is associated with better implant survival and patient-reported outcome measures (PROMs) albeit with a higher risk of periprosthetic fracture^10-12^.

It is important for surgeons and patients to know how the outcomes and risk profile of simultaneous bilateral UKAs compare with the traditionally established unilateral UKA procedure. The aim of this study was to compare the clinical outcomes of simultaneous bilateral UKAs with unilateral UKAs with key outcomes of interest being implant survival, revision indications, and patient survival. This is not a study of staged UKA procedures. We performed a matched study of simultaneous bilateral and unilateral medial Oxford UKAs. Our null hypothesis was no differences in implant survival or patient mortality between comparative groups.

## Material and Methods

### Data Sources and Linkage

**Fig. 1:**
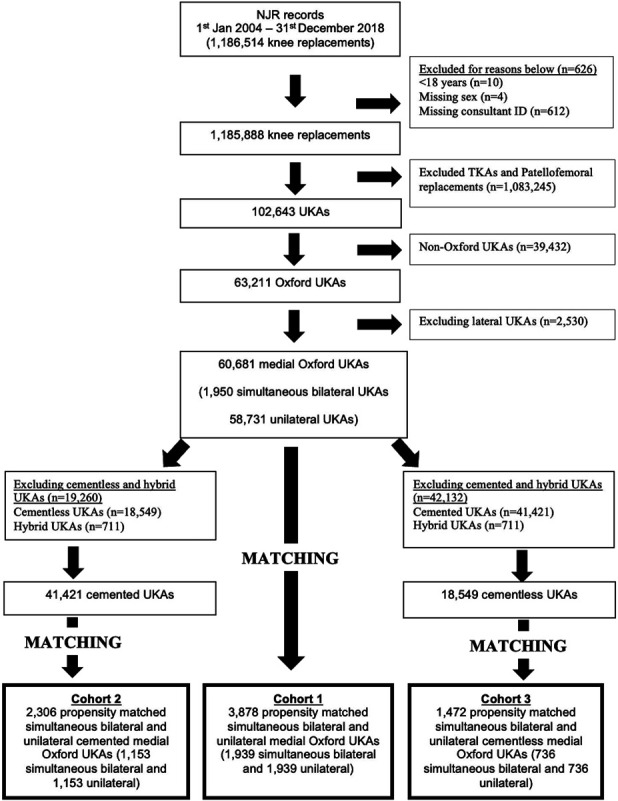
Data flowchart of NJR dataset cleaning and merging. NJR = National Joint Registry.

A retrospective observational study was performed using data from the National Joint Registry (NJR). The NJR is the world's largest arthroplasty register^13^. Between January 1, 2004, and December 31, 2018, 63,211 Oxford UKAs were performed. After removing lateral UKAs, there were 60,681 Oxford UKAs (1,950 simultaneous bilateral and 58,731 unilateral UKAs) available for matching (Fig. 1). Three matched cohorts were produced and were well balanced. The first cohort was to compare all unilateral and simultaneous bilateral UKAs (Fig. 1). The second was to compare all cemented unilateral and simultaneous bilateral UKAs (Fig. 1). The third was to compare all cementless unilateral and simultaneous bilateral UKAs (Fig. 1). Hybrid UKAs were not included in the cementless or the cemented cohorts.

Cohort 1 (all UKAs) consisted of 3,878 UKAs (1,939 unilateral and 1,939 simultaneous bilateral UKAs) (Table I). Cohort 2 (cemented UKAs) consisted of 2,306 UKAs (1,153 unilateral and 1,153 simultaneous bilateral cemented UKAs) (Appendix, Table S1). Cohort 3 (cementless UKAs) consisted of 1,472 UKAs (736 unilateral and 736 simultaneous bilateral cementless UKAs) (Appendix, Table S2).

**TABLE I:** Baseline Characteristics Before and After Matching Unilateral and Simultaneous Bilateral UKAs

	Unmatched			Matched		
	Unilateral UKAs (n = 58,731)	Simultaneous Bilateral UKAs (n = 1,950)	SMD	Unilateral UKAs (n = 1,939)	Simultaneous Bilateral UKAs (n = 1,939)	SMD
Covariate						
Age at surgery (yr)			0.02			0.01
Mean (SD)	64.8 (SD 9.5)	64.7 (SD 9.2)		64.8 (SD 9.6)	64.7 (SD 9.2)	
Gender (%)			0.14			0.01
Female	27,633 (47.1)	782 (40.1)		770 (39.7)	782 (40.3)	
Male	31,098 (52.9)	1,168 (59.9)		1,169 (60.3)	1,157 (59.7)	
BMI (kg/m^2^)			0.02			0.09
Mean (SD)	30.3 (SD 5.1, n = 38,928)	29.6 (SD 4.6, n = 1,388)		30.1 (SD 5.0, n = 1,318)	29.6 (SD 4.7, n = 1,378)	
Primary diagnosis (%)			0.13			0.04
Primary OA	58,107 (98.9)	1,942 (99.6)		1,935 (99.8)	1,931 (99.6)	
Other	624 (1.1)	8 (0.4)		4 (0.2)	8 (0.4)	
Fixation (%)			0.20			0.06
Cemented	40,267 (68.6)	1,154 (59.2)		1,184 (61.1)	1,152 (59.4)	
Cementless	17,786 (30.3)	763 (39.1)		735 (37.9)	754 (38.9)	
Hybrid	678 (1.1)	33 (1.7)		20 (1.0)	33 (1.7)	
ASA grade (%)			0.11			0.03
1	12,068 (20.6)	492 (25.2)		505 (26.0)	481 (24.8)	
2	41,481 (70.6)	1,294 (66.4)		1,263 (65.1)	1,294 (66.7)	
3 or above	5,182 (8.8)	164 (8.4)		171 (8.8)	164 (8.5)	
Year of surgery (%)			0.23			0.28
2004	1,491 (2.5)	32 (1.6)		50 (2.6)	32 (1.7)	
2005	1,877 (3.2)	64 (3.3)		48 (2.5)	64 (3.3)	
2006	2,687 (4.6)	60 (3.1)		75 (3.9)	60 (3.1)	
2007	3,525 (6.0)	72 (3.7)		136 (7.0)	72 (3.7)	
2008	3,967 (6.8)	162 (8.3)		119 (6.1)	160 (8.3)	
2009	4,123 (7.0)	130 (6.7)		133 (6.9)	130 (6.7)	
2010	3,948 (6.7)	186 (9.5)		130 (6.7)	186 (9.6)	
2011	4,017 (6.8)	162 (8.3)		119 (6.1)	162 (8.4)	
2012	4,128 (7.0)	180 (9.2)		125 (6.5)	179 (9.2)	
2013	3,859 (6.6)	118 (6.1)		135 (7.0)	118 (6.1)	
2014	4,466 (7.6)	134 (6.9)		157 (8.1)	131 (6.8)	
2015	4,719 (8.0)	136 (7.0)		146 (7.5)	136 (7.0)	
2016	5,066 (8.6)	156 (8.0)		187 (9.6)	153 (7.9)	
2017	5,536 (9.4)	156 (8.0)		192 (9.9)	154 (7.9)	
2018	5,322 (9.1)	202 (10.4)		187 (9.6)	202 (10.4)	
Surgeon caseload (UKAs/yr)	25.0 (SD 22.6)	37.2 (SD 26.8)	0.49	36.4 (SD 29.5)	36.8 (SD 26.5)	0.02
Chemical VTE prophylaxis (%)			0.23			0.03
None	4,726 (8.1)	212 (10.8)		224 (11.6)	207 (10.7)	
Aspirin	6,773 (11.5)	351 (18.0)		338 (17.4)	348 (18.0)	
LMWH	38,543 (65.6)	1,084 (55.6)		1,082 (55.8)	1,081 (55.8)	
Other	8,689 (14.8)	303 (15.6)		295 (15.2)	303 (15.6)	
Mechanical VTE prophylaxis (%)			0.06			0.02
Any	55,885 (95.2)	1,878 (96.3)		1,861 (96.0)	1,867 (96.3)	
None	2,846 (4.8)	72 (3.7)		78 (4.0)	72 (3.7)	

SMD = standardized mean differences, and UKA = unicompartmental knee arthroplasty.

### Statistical Analyses

There were differences in baseline characteristics between simultaneous bilateral and unilateral UKA groups (Table I, Appendix Tables S1, S2). Data were studied at the knee level. Propensity scores were generated through logistic regression, representing the probability that a patient received simultaneous bilateral UKAs and were generated from patient, implant, and surgical factors. The area under the receiver operating characteristic curve was 0.80 which is considered good for discrimination. Calibration plots showed good calibration of observed versus predicted risk across deciles of risk. All factors in Table I were used for matching except BMI. These factors are known to influence outcomes and hence were matched for^10,14-22^. BMI was not used for matching given missing data but was already well balanced between groups. This approach is well described^10,14,23-26^. Surgical factors included surgeon caseload, defined as the average number of UKAs performed annually^14,27^. We matched 1:1 on the logit of the propensity score using a 0.02-SD calliper width. Greedy matching without replacement was used given its superior performance for estimating treatment effects^28^. Standardized mean differences (SMDs) were examined to assess for any imbalance between groups, with SMDs of >10% suggestive of imbalance^29^. Outcomes of interest were (1) 10-year implant survival, (2) revision indications, and (3) mortality at 30 days, 90 days, 120 days, and 10 years postoperatively.

The Kaplan-Meier method was used to assess cumulative survival. The endpoint for implant survival was revision surgery (any implant component removed, exchanged, or added). The endpoint for mortality was death. The NJR still collects information for all patients who move within the United Kingdom, given it still captures data from all hospitals, but censors’ patients when they emigrate abroad. Survival rates were compared between groups, using Cox regression models, with the proportional hazards assumptions satisfied in all analyses. Hazard ratios (HRs) below 1 favor simultaneous bilateral UKAs. A multilevel frailty model was tested in the regression models to control for patient clustering within surgeons^12^. To account for clustering within the matched cohort, a robust variance estimator was used in regression models. The χ^2^ test with Yate correction was used to compare revision indications. 95% confidence intervals (CIs) are presented.

## Ethical Approval

Ethical approval from the South-Central Oxford B Research Ethics Committee (19/SC/0292) was obtained. Dataset linkage had Confidentiality Advisory Group (19/CAG/0054) approval.

## Source of Funding

The author or one or more of the authors have received or will receive benefits for personal or professional use from a commercial party related to the subject of this article. The commercial party played no role in the design, conduct, or interpretation of this study.

## Results

### All UKA Analyses—Cohort 1

The matched cohort consisted of 3,878 UKAs (1,939 unilateral and 1,939 simultaneous bilateral) (Table I). Patient, surgical (including caseload), and implant characteristics became well balanced after matching (Table I). Year of surgery had some residual imbalance but when adjusted for in the regression models did not alter the findings.

**Fig. 2:**
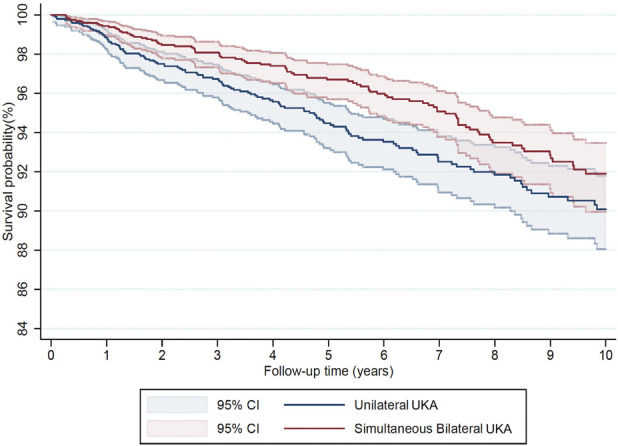
Kaplan-Meier comparison of implant survival of unilateral and simultaneous bilateral UKA. UKA = unicompartmental knee arthroplasty.

The mean follow-up for both groups was 6.3 years (SD 3.8). There were 137 (7.1%) revisions in the unilateral UKA group at a mean of 4.5 years postoperatively (SD 3.7) and 104 revisions (5.4%) in the simultaneous bilateral UKA group at a mean of 5.3 years postoperatively (SD 3.7). The 10-year implant survival in the unilateral UKA group was 90.1% (CI 88.1-91.8) and in the simultaneous bilateral UKA group was 91.9% (CI 90.0-93.5). The implant survival was significantly higher in the simultaneous bilateral UKA group with a HR 0.73 (CI 0.56-0.94, p = 0.01) (Fig. 2). Revisions for pain were significantly lower in the simultaneous bilateral UKA group (0.5% vs. 1.2%, p = 0.01). There were no other significant differences in revision indications between groups (Table II).

**TABLE II:** Revision Indications in Matched Cohort 1

Revision Indication	Unilateral UKAs (n = 1,939, %)	Simultaneous Bilateral UKAs (n = 1,939, %)	p
Aseptic loosening	26 (1.3)	29 (1.5)	0.68
OA progression	45 (2.3)	42 (2.2)	0.75
Pain*	23 (1.2)	9 (0.5)	0.01
Other	23 (1.2)	12 (0.6)	0.06
Dislocation subluxation revision	10 (0.5)	10 (0.5)	1.0
Instability	8 (0.4)	7 (0.4)	0.80
Component dissociation	5 (0.3)	2 (0.1)	0.26
Malalignment	5 (0.3)	4 (0.2)	0.74
Infection	6 (0.3)	5 (0.3)	0.76
Periprosthetic fracture	4 (0.2)	3 (0.2)	0.71
Lysis	2 (0.1)	5 (0.3)	0.26
Wear	7 (0.36)	7 (0.36)	1.0
Stiffness	0 (0	0 (0)	1.0

OA = osteoarthritis, and UKA = unicompartmental knee arthroplasty.

Comparisons between the frequency of revision indications were conducted using the χ^2^ test.

* denotes p < 0.05.

**Fig. 3:**
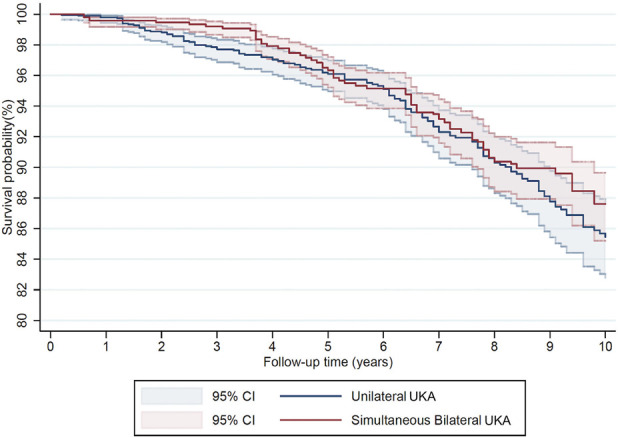
Kaplan-Meier comparison of patient survival of unilateral and simultaneous bilateral UKA. UKA = unicompartmental knee arthroplasty.

There was a total of 168 deaths (8.7%) in the unilateral UKA group at a mean of 6.4 (SD 3.6) postoperatively and 144 deaths (7.4%) in the simultaneous bilateral UKA group at a mean of 6.4 years (SD 3.0) postoperatively. Within the first 120 days of surgery, there were 2 deaths in the unilateral group and 0 deaths in the bilateral UKA group (Appendix, Table S3). The 10-year patient survival in the unilateral and simultaneous bilateral UKA groups was 85.5% (CI 82.8-87.7) and 87.6 (CI 85.2-89.7), respectively, with no significant differences between groups HR 0.83 (CI 0.66-1.04, p = 0.10) (Fig. 3).

### Cemented UKA Analyses—Cohort 2

The matched cohort of cemented unilateral and simultaneous bilateral groups consisted of 2,306 UKAs (1,153 unilateral and 1,153 simultaneous bilateral), and they were well balanced (Appendix, Table S1). There were 102 (8.9%) revisions in the unilateral and 90 (7.8%) in the bilateral UKA group. The mean follow-up was 8.2 years (SD 3.5) in both groups. The 10-year implant survival was 90.0% (CI 87.7%-92.0%) and 91.2% (CI 89.0-93.0), respectively, with a HR 0.85 (CI 0.64-1.14, p = 0.28) (Appendix, Figure S1). There were no differences in revision risk or revision indications between groups. There was a total of 153 (13.3%) and 115 (10.0%) deaths for the unilateral and simultaneous bilateral UKA groups. There were no deaths within 120 days of surgery. The 10-year patient survival in the unilateral and simultaneous bilateral cemented UKA groups was 84.4% (CI 81.5%-86.8%) and 88.0% (CI 85.5%-90.1%), respectively, with a HR 0.71 (CI 0.56-0.89, p = 0.004) (Appendix, Figure S2).

### Cementless UKA Analyses—Cohort 3

The matched cohort of cementless unilateral and simultaneous bilateral groups consisted of 1,472 UKAs (736 unilateral and 736 simultaneous bilateral), and they were well balanced (Appendix, Table S2). There were 19 (2.6%) revisions in the unilateral group and 12 (1.6%) in the bilateral UKA group. The mean follow-up was 3.6 years (SD 2.4) in both groups. The 10-year implant survival was 95.4% (CI 91.4%-97.6%) and 97.6% (CI 95.4-98.7), respectively, with a HR 0.66 (CI 0.32-1.37, p = 0.27) (Appendix, Figure S3). There were no differences in revision risk or revision indications between groups. There was a total of 26 (3.5%) and 20 (2.7%) deaths for the unilateral and simultaneous bilateral UKA groups. There were no deaths within 120 days of surgery. The 10-year patient survival in the unilateral and simultaneous bilateral UKA groups was 86.8% (CI 79.0%-91.8%) and 86.9% (CI 77.9%-92.4%), respectively, with a HR 0.84 (CI 0.47-1.51, p = 0.56) (Appendix, Figure S4).

## Discussion

This is the first study comparing the long-term outcomes of unilateral and simultaneous bilateral UKAs and is the largest comparative study. We found that for the overall analyses (cohort 1), simultaneous bilateral UKAs had better implant survival than unilateral UKAs and the revision rate for pain was reduced. There were no differences in postoperative mortality. On this basis, simultaneous bilateral Oxford UKAs should be considered to be at least as safe and effective as a single unilateral procedure. Therefore, in patients with severe symptoms from bilateral knee osteoarthritis, this study would suggest that bilateral simultaneous procedures probably would be advantageous compared with 2 unilateral procedures.

The revision rate, particularly for pain, of simultaneous bilateral UKAs was significantly lower than for unilateral UKAs. Previous comparative studies found no differences in revision rate but are limited by sample size^30,31^. It is unclear why the revision rate is lower. Marullo et al.^32^ compared gait parameters after unilateral and simultaneous bilateral UKAs and found significant improvement in multiple gait parameters after simultaneous bilateral procedures but not after unilateral procedures. A possible explanation is that bilateral UKA patients have to fully weight bear on both limbs and mobilize both limbs very soon after the surgery. It may be this early mobilization is important for avoiding ongoing pain and other problems. There may be other explanations, for example, patients having bilateral UKAs may be better candidates for an Oxford UKA satisfying the recommended indications, whereas this may not have been the case for all unilateral UKAs^33^.

We found no significant differences in short-term or long-term mortality between groups. Hao et al.^30^ did find evidence of greater metabolic upset in the early postoperative period following simultaneous bilateral UKAs but no increase in complications^30^. Feltri et al.^9^ found that simultaneous bilateral UKAs were not associated with any increase in mortality.

Subgroup analyses by fixation types found similar results with lower revision rates for simultaneous bilaterals than unilaterals in the cementless and cemented cohorts. However, the differences were not statistically significant. This is probably due to smaller number of cases in these analyses. The 10-year patient survival was also similar for unilateral and simultaneous bilateral UKAs in both overall analyses and cementless analyses. However, for the cemented analyses, the 10-year patient survival was significantly lower for unilateral compared with simultaneous bilateral UKAs. This is likely due to other comorbidities independent of the surgery.

There is concern about simultaneous bilateral procedures given the increased physiological insult. Our study has shown that simultaneous bilateral UKAs did not have a higher mortality. The benefits of simultaneous bilateral UKAs do not appear to apply to TKA. Memtsoudis et al.^34^ found that patients undergoing bilateral TKAs had over double the mortality risk when compared with unilateral TKA. Coupled with evidence that TKA has higher mortality risk than UKA it may not be as well suited to bilateral procedures as UKA^1^. This is probably because UKA is a smaller operation and performed minimally invasively. However, our study had generally healthy patients with only about 8% being ASA 3 or above, whereas TKA often has a greater proportion of higher ASA patients.

## Limitations

Our study is based on observational data from the joint registry, and there may be unexplored confounders. We did not have radiographic data so could not assess the severity of the preoperative arthritis and did not have PROMs. It is not possible to confirm causality in registry-based studies. We did not have data on ethnicity or race which may affect generalizability. There was a substantial proportion of BMI data missing, so we did not match on BMI. However, the BMI distribution between groups was well balanced. The only way to achieve balance with respect to both known and unknown confounders is with a randomized trial. However, to compare revision rates would require very large numbers and long follow-up, which would be impractical; propensity matching offers the next best alternative.

## Conclusion

In the overall analyses, the risk of revision, particularly revision for pain, is lower in simultaneous bilateral UKAs compared with unilateral UKA. There was no increase in mortality when performing simultaneous bilateral UKAs. These results should encourage surgeons to perform simultaneous bilateral UKAs in cases with bilateral unicompartmental arthritis to allow patients to benefit from a reduced number of anesthetics, operations, and postoperative recoveries.

## Appendix

Supporting material provided by the authors is posted with the online version of this article as a data supplement at jbjs.org (http://links.lww.com/JBJSOA/B64). This content was not copyedited or verified by JBJS.

## References

[1] Liddle AD Judge A Pandit H Murray DW. Adverse outcomes after total and unicompartmental knee replacement in 101 330 matched patients: a study of data from the National Joint Registry for England and Wales. Lancet. 2014;384(9952):1437-45. 25012116 10.1016/S0140-6736(14)60419-0

[2] Mohammad HR Judge A Murray DW. A matched comparison of the patient-reported outcome measures of 38,716 total and unicompartmental knee replacements: an analysis of linked data from the National Joint Registry of England, Northern Ireland and Isle of Man and England's National PROM collection programme. Acta Orthopaedica. 2021;92(6):701-8. 34309481 10.1080/17453674.2021.1956744 PMC8635544

[3] Mohammad HR Liddle AD Judge A Murray DW. A matched comparison of long-term outcomes of total and unicompartmental knee replacements in different ages based on national databases: analysis of data from the National Joint Registry for England, Wales, Northern Ireland, and the Isle of Man. J Arthroplasty. 2022;37(2):243-51. 34619307 10.1016/j.arth.2021.09.018

[4] Meehan JP Danielsen B Tancredi DJ Kim S Jamali AA White RH. A population-based comparison of the incidence of adverse outcomes after simultaneous-bilateral and staged-bilateral total knee arthroplasty. J Bone Jt Surg. 2011;93(23):2203-13. 10.2106/JBJS.J.01350 22159856

[5] Sayeed SA Sayeed YA Barnes SA Pagnano MW Trousdale RT. The risk of subsequent joint arthroplasty after primary unilateral total knee arthroplasty, a 10-year study. J Arthroplasty. 2011;26(6):842-6. 20884167 10.1016/j.arth.2010.08.016

[6] Lewin AM Cashman K Harries D Ackerman IN Naylor JM Harris IA. First knee for pain and function versus second knee for quality of life: a registry study of patient-reported outcomes following staged bilateral knee arthroplasty in Australia. Bone Jt Open. 2024;5(3):202-9. 38461859 10.1302/2633-1462.53.BJO-2023-0035.R1 PMC10924693

[7] Hu J Liu Y Lv Z Li X Qin X Fan W. Mortality and morbidity associated with simultaneous bilateral or staged bilateral total knee arthroplasty: a meta-analysis. Arch Orthop Trauma Surg. 2011;131(9):1291-8. 21359869 10.1007/s00402-011-1287-4

[8] Fu D Li G Chen K Zeng H Zhang X Cai Z. Comparison of clinical outcome between simultaneous-bilateral and staged-bilateral total knee arthroplasty: a systematic review of retrospective studies. J Arthroplasty. 2013;28(7):1141-7. 23518424 10.1016/j.arth.2012.09.023

[9] Feltri P Mondini Trissino da Lodi C Grassi A Zaffagnini S Candrian C Filardo G. One-stage bilateral unicompartmental knee arthroplasty is a suitable option vs. the two-stage approach: a meta-analysis. EFORT Open Rev. 2021;6(11):1063-72. 34909225 10.1302/2058-5241.6.210047 PMC8631243

[10] Mohammad HR Matharu GS Judge A Murray DW. Comparison of the 10-year outcomes of cemented and cementless unicompartmental knee replacements: data from the National Joint Registry for England, Wales, Northern Ireland and the Isle of Man. Acta Orthop. 2020;91(1):76-81. 31635503 10.1080/17453674.2019.1680924 PMC7006803

[11] Mohammad HR Barker K Judge A Murray DW. A comparison of the periprosthetic fracture rate of unicompartmental and total knee replacements: an analysis of data of> 100,000 knee replacements from the National Joint Registry for England, Wales, Northern Ireland and the Isle of Man and Hospital Episode Statistics. J Bone Jt Surg. 2023;105(23):1857-66. 10.2106/JBJS.22.01302 37733918

[12] Mohammad HR Judge A Murray DW. A matched comparison of implant and functional outcomes of cemented and cementless unicompartmental knee replacements: a Study from the national joint registry for England, Wales, Northern Ireland and the Isle of Man and the hospital episode statistics patient reported outcome measures database. J Bone Jt Surg. 2024;106(17):1553-62. 10.2106/JBJS.23.01418 38980924

[13] National Joint Registry. National Joint Registry 15th Annual Report. National joint registry for England, Wales, Northern Ireland and Isle of Man. 2018.

[14] Mohammad HR Matharu GS Judge A Murray DW. The effect of surgeon caseload on the relative revision rate of cemented and cementless unicompartmental knee replacements: an analysis from the national joint registry for England, Wales, Northern Ireland and the Isle of Man. J Bone Jt Surg. 2020;102(8):644-53. 10.2106/JBJS.19.01060 32004190

[15] Bayliss LE Culliford D Monk AP Glyn-Jones S Prieto-Alhambra D Judge A Cooper C Carr AJ Arden NK Beard DJ Price AJ. The effect of patient age at intervention on risk of implant revision after total replacement of the hip or knee: a population-based cohort study. Lancet. 2017;389(10077):1424-30. 28209371 10.1016/S0140-6736(17)30059-4 PMC5522532

[16] Murphy BPd Dowsey M Spelman T Choong P. The impact of older age on patient outcomes following primary total knee arthroplasty. Bone Jt J. 2018;100-B(11):1463-70. 10.1302/0301-620X.100B11.BJJ-2017-0753.R6 30418062

[17] Lim JBT Chi CH Lo LE Lo WT Chia S-L Yeo SJ Chin PL Tay KJD Lo NN. Gender difference in outcome after total knee replacement. J Orthop Surg. 2015;23(2):194-7. 10.1177/230949901502300216 26321549

[18] Hamilton T Pandit H Lombardi A Adams J Oosthuizen C Clavé A Dodd CAF Berend KR Murray DW. Radiological decision aid to determine suitability for medial unicompartmental knee arthroplasty: development and preliminary validation. Bone Jt J. 2016;98-B(10_suppl_B):3-10. 10.1302/0301-620X.98B10.BJJ-2016-0432.R1 PMC5047136 27694509

[19] Asa grading vs. mortality in elective orthopaedic procedures. In: Prempeh E Cherry R, eds. Orthopaedic Proceedings. The British Editorial Society of Bone and Joint Surgery; 2008:536.

[20] Elmallah RD Cherian JJ Robinson K Harwin SF Mont MA. The effect of comorbidities on outcomes following total knee arthroplasty. J Knee Surg. 2015;28(05):411-6. 25892005 10.1055/s-0035-1549023

[21] Lenguerrand E Whitehouse MR Beswick AD Kunutsor SK Foguet P Porter M Blom AW. Risk factors associated with revision for prosthetic joint infection following knee replacement: an observational cohort study from England and Wales. Lancet Infect Dis. 2019;19(6):589-600. 31005559 10.1016/S1473-3099(18)30755-2 PMC6531378

[22] Selby R Borah BJ McDonald HP Henk HJ Crowther M Wells PS. Impact of thromboprophylaxis guidelines on clinical outcomes following total hip and total knee replacement. Thromb Res. 2012;130(2):166-72. 22365491 10.1016/j.thromres.2012.01.013

[23] Mohammad HR Matharu GS Judge A Murray DW. A matched comparison of revision rates of cemented Oxford unicompartmental knee replacements with single and twin peg femoral components, based on data from the National Joint Registry for England, Wales, Northern Ireland and the Isle of Man. Acta Orthop. 2020;91(4):420-5. 32420778 10.1080/17453674.2020.1748288 PMC8023905

[24] Mohammad HR Matharu GS Judge A Murray DW. New surgical instrumentation reduces the revision rate of unicompartmental knee replacement: a propensity score matched comparison of 15,906 knees from the National Joint Registry. Knee. 2020;27(3):993-1002. 32115338 10.1016/j.knee.2020.02.008

[25] Matharu GS Judge A Murray DW Pandit HG. Trabecular metal acetabular components reduce the risk of revision following primary total hip arthroplasty: a propensity score matched study from the National Joint Registry for England and Wales. J Arthroplasty. 2018;33(2):447-52. 28947370 10.1016/j.arth.2017.08.036

[26] Matharu GS Judge A Murray DW Pandit HG. Outcomes after metal-on-metal hip revision surgery depend on the reason for failure: a propensity score-matched study. Clin Orthop Relat Res. 2018;476(2):245-58. 29529653 10.1007/s11999.0000000000000029 PMC6125743

[27] Liddle AD Pandit H Judge A Murray DW. Effect of surgical caseload on revision rate following total and unicompartmental knee replacement. J Bone Jt Surg. 2016;98(1):1-8. 10.2106/JBJS.N.00487 26738897

[28] Austin PC. Some methods of propensity‐score matching had superior performance to others: results of an empirical investigation and Monte Carlo simulations. Biometrical J. 2009;51(1):171-84. 10.1002/bimj.200810488 19197955

[29] Austin PC. Balance diagnostics for comparing the distribution of baseline covariates between treatment groups in propensity‐score matched samples. Stat Med. 2009;28(25):3083-107. 19757444 10.1002/sim.3697 PMC3472075

[30] Hao Y Li J Li J Zhao F Yu X Liang S Zhang C Dong W Liu G. Comparison of clinical outcomes of bilateral and unilateral unicompartmental knee arthroplasty for the treatment of knee osteoarthritis. Sci Rep. 2024;14(1):30953. 39730682 10.1038/s41598-024-81995-7 PMC11680845

[31] Chen J Lo N Jiang L Chong H Tay D Chin P Chia SL Yeo SJ. Simultaneous versus staged bilateral unicompartmental knee replacement. Bone Jt J. 2013;95-B(6):788-92. 10.1302/0301-620X.95B6.30440 23723273

[32] Marullo M Vitale JA Stucovitz E Romagnoli S. Simultaneous bilateral unicompartmental knee replacement improves gait parameters in patients with bilateral knee osteoarthritis. Knee. 2019;26(6):1413-20. 31537415 10.1016/j.knee.2019.08.014

[33] Pandit H Hamilton T Jenkins C Mellon S Dodd C Murray D. The clinical outcome of minimally invasive phase 3 Oxford unicompartmental knee arthroplasty: a 15-year follow-up of 1000 UKAs. Bone Jt J. 2015;97-B(11):1493-9. 10.1302/0301-620X.97B11.35634 26530651

[34] Memtsoudis SG Ma Y González Della Valle A Mazumdar M Gaber-Baylis LK MacKenzie CR Sculco TP. Perioperative outcomes after unilateral and bilateral total knee arthroplasty. Anesthesiology. 2009;111(6):1206-16. 19934863 10.1097/ALN.0b013e3181bfab7d PMC2803038

